# Identification of prognostic biomarkers related to epithelial-mesenchymal transition and anoikis in hepatocellular carcinoma using transcriptomics and single-cell sequencing

**DOI:** 10.3389/fcell.2025.1600546

**Published:** 2025-06-19

**Authors:** Maobing Wang, Lu Cheng, Kuo Qi, Haiping Wang, Xun Li

**Affiliations:** ^1^ The First School of Clinical Medicine, Lanzhou University, Lanzhou, China; ^2^ Department of General Surgery, The First Hospital of Lanzhou University, Lanzhou, Gansu, China; ^3^ Gansu Provincial Key Laboratory of Biotherapy and Regenerative Medicine, Lanzhou, Gansu, China; ^4^ Gansu Institute of Hepatobiliary and Pancreatic Surgery, Lanzhou, Gansu, China; ^5^ Gansu Province General Surgery Clinical Medical Research Center, Lanzhou, Gansu, China

**Keywords:** hepatocellular carcinoma, epithelial mesenchymal transition, anoikis, single-cell RNA sequencing, biomarkers

## Abstract

**Background:**

Epithelial-mesenchymal transition (EMT) and anoikis are critically associated with hepatocellular carcinoma (HCC). However, the precise mechanisms underlying their roles in HCC remain unclear. This study aims to explore the involvement of EMT-related genes (EMTRGs) and anoikis-related genes (ARGs) in HCC.

**Methods:**

Data from TCGA-HCC, ICGC-LIPI - JP, GSE149614, EMTRGs and ARGs were utilised in this study. It utilised single-cell RNA sequencing for cell sorting. Biomarkers were identified through analyses such as differential expression analysis and weighted gene co-expression network analysis (WGCNA). The risk model and nomogram were constructed based on biomarkers. Subsequently, the potential functions of biomarkers were explored through methods such as enrichment analysis and immune microenvironment analysis. Finally, to confirm the expression of these biomarkers in different prognostic groups, gene expression levels were quantified using real-time quantitative polymerase chain reaction (RT-qPCR).

**Results:**

LAMA4, C7, KPNA2, STMN1, and SF3B4 were identified as biomarkers. The risk score emerged as an independent prognostic factor for patients with HCC. The nomogram showed that these five biomarkers had good predictive ability for the 1-, 3-, and 5-year survival rates of HCC patients. Drug sensitivity analysis revealed significant associations between the IC50 values of 23 drugs and risk scores. In the GSE149614 dataset, most biomarkers were predominantly expressed in stromal cells (endothelial cells and fibroblasts). In TCGA-HCC, all genes, except C7, were upregulated in the HCC samples. RT-qPCR analysis revealed statistically significant upregulation of STMN1 and SF3B4 transcripts in the HCC group, consistent with TCGA-HCC dataset.

**Conclusion:**

This study identified five EMTRGs and ARGs (LAMA4, C7, KPNA2, STMN1, and SF3B4) as biomarkers of HCC, offering new insights for further research in HCC pathogenesis.

## 1 Introduction

Hepatocellular carcinoma (HCC) is the most prevalent form of primary liver cancer and poses a major global health threat (Hamaya et al., 2023), with high incidence rates (Liu H. et al., 2024) and accounting for 7.8% of cancer-related deaths annually (Bray et al., 2024). HCC is associated with a poor prognosis, as the 5-year survival rate for patients in advanced stages is a mere 10%–20% (Laube et al., 2020). Surgical resection remains the gold standard treating early-stage HCC, significantly improving long-term survival and offering potential for a cure. In contrast, treatment for advanced HCC typically involves conversion therapy, including local treatments such as transarterial chemoembolization(TACE) and hepatic arterial infusion chemotherapy (HAIC), as well as systemic approaches like targeted therapies combined with immunotherapy and combinations of local and systemic therapies (Zhou and Song, 2021). However, approximately two-thirds of patients with HCC are diagnosed at stages III or IV, where treatment efficacy is limited (ECTRX, 2017). A key challenge in managing HCC is the identification and validation of biomarkers that can enable early intervention and improve therapeutic outcomes (Sangro et al., 2021). This study aims to identify novel molecular biomarkers for early diagnosis, thereby enhancing the therapeutic efficacy of HCC.

Epithelial-mesenchymal transition (EMT) is a developmental and pathological process in which epithelial cells undergo a phenotypic conversion to mesenchymal cells, acquiring migratory and invasive properties (Grigore et al., 2016). EMT plays a pivotal role in cancer metastasis (Jehanno et al., 2022), promoting tumor cell proliferation, reducing apoptosis and senescence, and enhancing immune evasion (Chanvorachote et al., 2022). EMT-related molecular markers have been associated with poor prognosis in patients with HCC (Li et al., 2024). For instance, Zhang et al. demonstrated that AHSA1 facilitates proliferation and EMT through the ERK/CALD1 signaling pathway, leading to disease progression and adverse outcomes (Zhang J. et al., 2022). Alterations in ZEB-1 and E-cadherin expression are likely drives of HCC progression through EMT (Hashiguchi et al., 2013). Anoikis is a distinct form of cell death that occurs when cells lose attachment to the extracellular matrix (ECM) and neighboring cells, triggering a signaling cascade that leads to cell apoptosis (Taddei et al., 2012). Tumor cells can develop resistance to anoikis when detached from the ECM, reducing their susceptibility to cell death, thereby facilitating tissue invasion and metastasis (Chen et al., 2023). Zhu et al. found that lncRNA signatures associated with anoikis could serve as prognostic biomarkers for HCC, with implications for immune infiltration (Zhu et al., 2024). EMT and anoikis are closely linked, playing critical roles in the invasive phase of primary tumor growth (Cao et al., 2016). Abnormal expression of miR-424-5p promotes anoikis resistance and EMT, advancing HCC metastasis by enhancing tumor cell survival and invasion, while its downregulation accelerates HCC progression (Zhang et al., 2014). Additionally, increased miR-450a expression inhibits EMT and promotes anoikis in ovarian cancer cells, reducing invasiveness and migration (Muys et al., 2019). Beyond cancer, EMT and anoikis are implicated in other diseases. In Crohn’s disease, defective matrix remodeling may trigger perianal fistulas via mechanical activation of EMT (Rizzo et al., 2023), and molecules regulating anoikis may serve as therapeutic targets for colorectal cancer (Liu et al., 2023). However, the relationship between EMT, anoikis and HCC remains underexplored.

In this study, a predictive model for HCC prognosis was constructed using EMT-related genes (EMTRGs) and anoikis-related genes (ARGs) through bioinformatics. Additionally, variations in immune infiltration and chemosensitivity were analyzed. This work provides a theoretical foundation and new insights into the molecular mechanisms of HCC, with implications for predicting outcomes, and developing targeted and immunotherapeutic strategies.

## 2 Materials and methods

### 2.1 Data source

The Cancer Genome Atlas (TCGA)-HCC dataset, was retrieved from the TCGA database (http://cancergenome.nih.gov), comprising transcriptomic, clinical, survival, and mutation data from 363 HCC samples and 49 normal samples. A total of 360 HCC samples with survival data were used as the training set. The HCC-related ICGC-LIPI-JP dataset was sourced from the International Cancer Genome Consortium (ICGC) database (https://dcc.icgc.org/releases/current/Projects/LIRI-JP), containing 232 HCC samples for verification. The GSE149614 single-cell dataset (platform GPL24676) was obtained from the Gene Expression Omnibus (GEO) database (https://www.ncbi.nlm.nih.gov/), which includes 10 HCC samples and 8 normal samples. The 200 EMTRGs and 35 ARGs were obtained from the published literature (Zhou et al., 2023; Cai and Zhou, 2022).

### 2.2 Single-cell RNA-seq analysis and difference analysis

To elucidate the cellular mechanisms underlying HCC, analyses were performed on the GSE149614 dataset using the ‘Seurat’ package (v 4.0.5). Cells with fewer than three cells or 10 features were excluded, as were data with RNA counts below 4,000, RNA feature counts between 100 and 8,000, and mitochondrial content exceeding 3% (Satija et al., 2015). Data were normalized using NormalizeData, and the FindVariableFeatures function was used to identify genes exhibiting high intercellular variability (vst = 2000). Canonical correlation analysis (CCA) was employed to correct for batch effects, followed by conducted principal component analysis (PCA). Unsupervised clustering was carried out with the FindNeighbors and FindClusters functions in Seurat, and clusters were visualized using t-SNE. Positive marker genes were identified using the FindAllMarkers function with parameters min. pct = 0.6, only. pos = TRUE, and threshold = 0.5. These markers were integrated with the CellMarker database to determine cell subpopulations, which were further validated using the SingleR algorithm (Aran et al., 2019). The differentially expressed genes 1 (DEGs1) in different cell types in HCC samples and control samples were screened through the FindMarker function (*p*-value <0.05, |log_2_ fold change (FC)| ≥ 0.5). Cell-cell communication within the GSE149614 dataset was assessed using the ‘CellChat’ package (Jin et al., 2021) (v 1.6.1), with a focus on ligand-receptor signaling differences between HCC and normal subpopulations. DEGs2 in the TCGA-HCC dataset were identified using DESeq2 (v 1.36.1) comparing HCC samples with normal tissues (|log_2_FC| > 0.5, p adj <0.05) (Love et al., 2014).

### 2.3 Weighted gene co-expression network analysis (WGCNA)

To calculate EMT and anoikis scores, the GSVA package (v 1.46.0) was used in the TCGA-HCC dataset, providing trait data for WGCNA (Hänzelmann et al., 2013). The WGCNA package (v 1.70–3) was employed to construct the co-expression network (Langfelder and Horvath, 2008). Specifically, hierarchical clustering was first performed using euclidean distances of expression to check for outliers in the samples, and outlier samples were removed. Then, the optimal soft threshold was set based on the scale-free fit index (signed *R* ^2^) and average connectivity (close to 0). A clustering tree was built based on similarity and adjacency calculations. And the dynamic tree cutting algorithm was used to segment the network into modules, with a minimum of 100 genes per module. Modules most significantly associated with EMT and anoikis scores were defined as key modules. Genes were selected as key module genes if they satisfied the criteria of |gene significance (GS)| > 0.4 and |module membership (MM)| > 0.634 (Chen et al., 2019).

### 2.4 Functional analysis and construction of protein-protein interaction network (PPI)

Candidate genes were identified by filtering those that overlapped with key module genes, DEGs1, and DEGs2 based on the results of the previous analyses. To explore the cellular functions and associated pathways of these candidate genes. Gene Ontology (GO) and Kyoto Encyclopedia of Genes and Genomes (KEGG) pathway enrichment analyses were performed using the clusterProfiler package (v 4.4.4) and the human gene annotation package org. Hs.e.g.,.db (v 3.15.0) (p.adj <0.05) (Yu et al., 2012). The PPI network for these candidate genes was constructed using the STRING database (http://string.embl.de/) with a confidence level set at 0.4.

### 2.5 Creation of the risk model

The expression data of the candidate genes were extracted and combined with overall survival (OS) information from patients with HCC. In the training set, to identify biomarkers associated with HCC prognosis, univariate Cox regression analysis was performed using the survival package (v-3.3–1) (Ramsay et al., 2018) (*p*-value <0.05, hazard ratio (HR) ≠ 1), followed by least absolute shrinkage and selection operator (LASSO) regression from the glmnet package (v 4.1–1) (Li et al., 2022). In the LASSO analysis, the parameter “family” was set as Cox, and ten-fold cross-validation was performed. Biomarkers were screened according to the lambda min value. Using the gene expression data of the TCGA-HCC training set and the coefficients of biomarkers obtained from LASSO analysis, a prognostic risk model was developed. The risk score formula was as follows: $\text{risk score}=\sum_{i=1}^{n}\text{coef}\left(\text{genei}\right)*\text{expr}\left(\text{genei}\right)$ , where coef represents the coefficient of each gene derived from LASSO analysis, derived from stepwise regression, reflecting the impact of individual gene expression levels on the overall risk score, and expr denotes the expression level of the *i*th gene.

Based on the optimal cutoff value of the risk scores, patients in the training set were categorized into two risk groups (High-risk group and low-risk group). Survival differences between these groups were assessed using Kaplan-Meier (K-M) curves generated with the survminer package (v 0.4.9) (*p*-value <0.05). The performance of the prognostic model was evaluated using receiver operating characteristic (ROC) curves from the survivalROC package (v 1.0.3) (Area Under Curve (AUC) > 0.6) (Heagerty et al., 2000). The model was further validated in an independent validation set by same method.

### 2.6 Creation of the nomogram

In the TCGA-HCC dataset, univariate Cox regression analysis (*p*-value <0.05, HR ≠ 1) and Proportional Hazards (PH) assumption tests (*p*-value >0.05) were performed on clinicopathological factors such as risk score, grade, TNM stage, and stage. Following univariate Cox analysis and PH test, multivariate Cox analysis (with HR ≠ 1 and P < 0.05) was performed to determine the independent prognostic factors among the variables that met the criteria. A nomogram incorporating the identified biomarkers was developed using the ‘rms’ package (v 6.2–0) (Sachs, 2017). In this nomogram, each factor was assigned a specific number of ‘Points,’ and the cumulative sum of these points, termed ‘Total Points,’ was used to predict the 1-, 3-, and 5-year survival probabilities for patients with HCC. A higher ‘Total Points’ score indicated a worse survival prognosis. The predictive accuracy of the nomogram, was assessed using a calibration curve, which visually compared the predicted and observed survival rates for patients with HCC. Additionally, ROC curves were concurrently for the 1-, 3-, and 5-year survival predictions to evaluate the nomogram’s discriminative ability. To assess its clinical utility, decision curve analysis (DCA) was performed.

### 2.7 Enrichment analysis

Further analyses were conducted to examine the characteristics of the biomarkers. The correlation of these biomarkers with other genes in the TCGA-HCC dataset was analyzed, followed by sorting the genes’ coefficients by magnitude. Gene Set Enrichment Analysis (GSEA) was performed using the HALLMARK dataset through the “clusterProfiler” package (v 4.4.4) and “org.Hs.e.g.,.db” package (v 3.15.0) (Wu et al., 2021).

### 2.8 Immune characteristic analysis

To explore the variations in the microenvironment between risk groups, the immune, matrix, and ESTIMATE scores were calculated for all HCC samples in the TCGA-HCC dataset using the ESTIMATE algorithm. The abundance of 24 immune cell types and 9 immune-related pathways was determined in all TCGA-HCC samples using the single-sample GESA (ssGSEA) algorithm. TIDE score and immune treatment responses for each HCC sample were retrieved from the TIDE database (hhttp://tide.dfci.harvard.edu/). Differences in the aforementioned scores, immune cell types, and immune-related pathways between the risk groups were compared. Spearman correlation analyses were performed to assess the relationships between biomarkers and differentially infiltrating immune cells, as well as between biomarkers and immune-related pathways (|co)| > 0.3, *p*-value <0.05).

### 2.9 Gene mutation analysis

Tumor mutation burden (TMB) was calculated for each HCC sample using mutation data from the TCGA database. To examine genetic differences between the risk groups, TCGA-HCC individuals were stratified into high-TMB (H-TMB) and low TMB (L-TMB) groups based on the optimal threshold determined by survminer for survival difference analysis. Mutation analysis was conducted using the maftools package (v 2.6.05), and somatic interactions were assessed to identify correlations between mutated genes (Mayakonda et al., 2018).

### 2.10 Drug sensitivity analysis and molecular docking

To explore the potential application of common chemotherapy drugs for patients with different risk profiles, 138 chemotherapy drugs were obtained from the GDSC database (http://cancerrxgene.org). IC50 values were computed for each drug in every HCC sample using the pRRophetic package (v 0.5) (Jiang et al., 2022). Additionally, the correlation analysis was conducted between the risk score and the IC50 of 138 drugs (|cor| ≥ 0.5, *p*-value <0.05). The drug exhibiting the strongest correlation with the biomarkers was selected for further correlation analysis. Subsequently, the most correlated drug-biomarker pair was chosen for molecular docking studies. The 3D conformer structure of the drug was downloaded from the PubChem database (https://pubchem.ncbi.nlm.nih.gov/), and the crystal structures of biomarkers were retrieved from the Protein Data Bank (PDB) (https://www1.rcsb.org/) database. Molecular docking simulations were carried out with AutoDock Vina, and the results were visualized using PyMol software.

### 2.11 Expression analysis

Further analysis examined the expression of biomarkers across different cell subpopulations. The Wilcoxon rank-sum test was performed to compare the expression levels of biomarkers between HCC samples and normal samples in the TCGA-HCC and GSE76427 datasets. The Additionally, 10 frozen tissue samples were obtained from 5 patients with HCC and 5 normal individuals at the First Hospital of Lanzhou University (ethics approval number: LDYYLL-2024–716). RNA was extracted from 50 mg of each tissue sample using TRIzol (Ambion, Austin, United States). The purity and concentration of 1 µL of RNA were measured using a NanoDrop spectrophotometer. Following the instructions of the SureScript-First-strand cDNA-Synthesis-Kit, mRNA was reverse-transcribed into cDNA. The CFX96 real-time quantitative PCR system was used for 40 cycles of amplification, and the amplification and dissolution curves were prepared. Relative gene expression was calculated using the 2^–△△Ct^ method, with statistical significance determined by GraphPad Prism 5.

### 2.12 Statistical analysis

**FIGURE 1:**
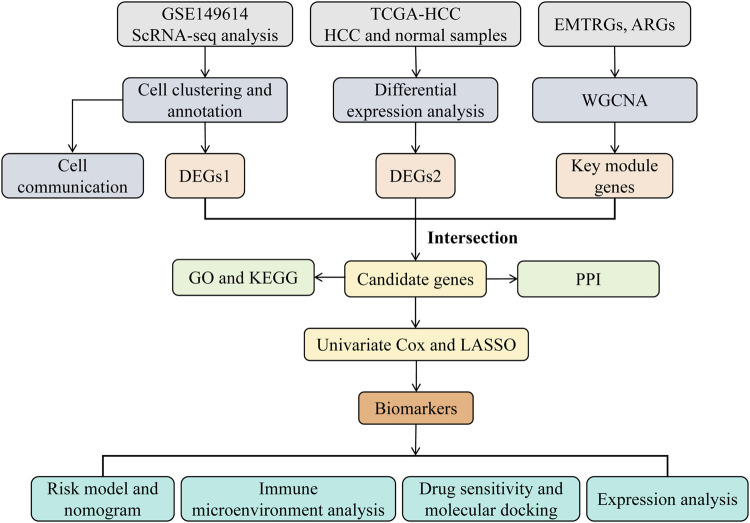
The analysis flowchart of this study.

Data analysis was performed using the R statistical software (v 4.2.2). Group differences were evaluated using the Wilcoxon rank-sum test, with a significance threshold of *p*-value <0.05. The overall analysis process of this study was shown in Figure 1.

## 3 Results

### 3.1 There were 2,804 DEGs1 in GSE149614 and 4,202 DEGs2 in TCGA-HCC

**FIGURE 2:**
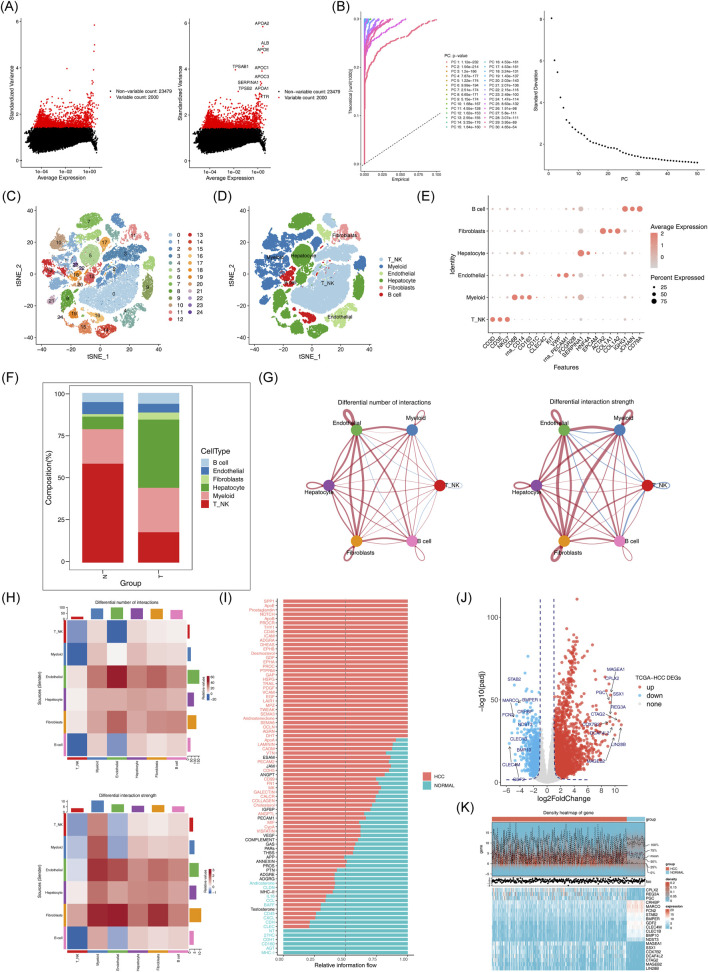
Acquisition of differentially expressed genes and processing of single cell sequencing data. **(A)** Expression of highly variable genes across cells. **(B)** Examination and visualization of PCA results using ElbowPlot. **(C)** Cell clustering can be visualized by t-SNE. **(D)** t-SNE clustering distribution of cell subsets. **(E)** Marker gene expression plot for each cluster. **(F)** Histogram of cell proportions. **(G-H)**: Differences in the number and intensity of interactions between different cell populations in the communication networks across groups. **(I)** Signal pathway differences between groups. **(J)** Volcano plot of differentially expressed genes between HCC and normal samples. **(K)** Expression heatmap of the top 10 downregulated differentially expressed genes in HCC versus normal samples.

The scRNA-seq analysis of the GSE149614 dataset was performed to investigate the cellular mechanisms underlying HCC. Following quality control, the dataset was deemed suitable for further analysis (Supplementary Figure S1). After cell screening and standardization, 2,000 highly variable genes were identified (Figure 2A). The first 30 principal components (PCs) at the inflection point were selected for subsequent analysis (Figure 2B). Unsupervised clustering of the cells resulted in the identification of 25 distinct clusters (Figure 2C). These clusters were further classified into six cell subpopulations based on marker gene expression: NK_T cell, Hepatocyte, Myeloid, B cell, Endothelial, and Fibroblast (Figures 2D, E). Significant differences in the proportions of these cell subpopulations between HCC and normal samples were observed (Figure 2F). A total of 2,804 differentially expressed genes (DEGs1) were identified in GSE149614 between HCC and normal groups (Supplementary Table S1). In cell-cell communication analysis, endothelial cells, hepatocytes, fibroblasts, myeloid cells, and B cells exhibited increased interaction numbers and strength in the HCC group (Figures 2G, H). Moreover, the ligand-receptor signaling differences between HCC and normal samples showed that pathways such as SPP1, LAMININ and VTN were more prevalent in the HCC microenvironment, suggesting enhanced tumor growth, invasion, and immune evasion. In contrast, immune-related signaling pathways like MHC-I, CXCL and CCL were more prominent in the normal samples (Figure 2I). Additionally, 4,202 DEGs2 (3,039 upregulated and 1,163 downregulated) were identified in thr TCGA-HCC dataset (Figures 2J, K).

### 3.2 The 3,157 key module genes were obtained by WGCNA

**FIGURE 3:**
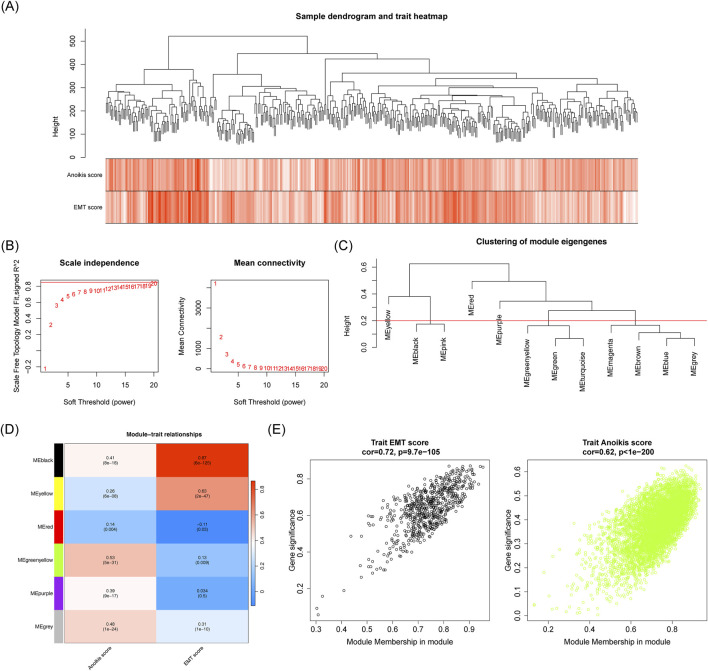
Identification of associated with EMT and anoikis scores. **(A)** Sample clustering and phenotypic heatmap. **(B)** Soft threshold filtering. **(C)** Module clustering tree. **(D)** Correlation heatmap of modules with EMT and anoikis scores. **(E)** Scatter plot showing the correlation between MEblack/MEgreenyellow module genes and EMT score and anoikis score traits.

No outliers were detected among the HCC samples in the TCGA-HCC dataset (Figure 3A). When β was set to 20 and *R* ^2^ = 0.85, the mean connectivity converged to 0 (Figure 3B). Six co-expression modules were identified, with the MEblack module showing the strongest correlation with EMT score (cor = 0.87, *p* < 0.05) and the MEgreenyellow module correlating strongly with the anoikis score (cor = 0.53, *p* < 0.05) (Figures 3C, D). Based on criteria |GS| > 0.4 and |MM| > 0.6, a total of 3.157 key module genes were identified (Figure 3E).

### 3.3 The 82 candidate genes enriched in many pathways

**FIGURE 4:**
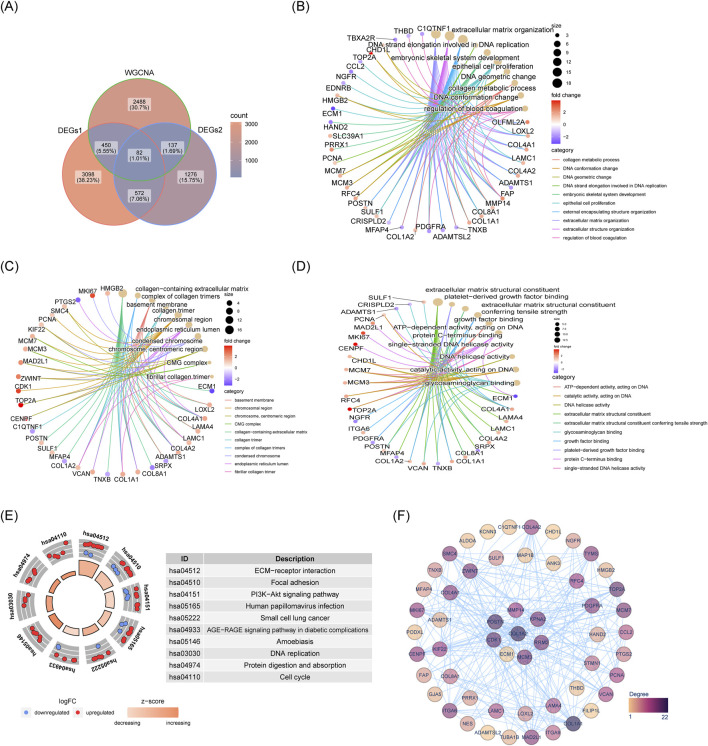
Acquisition and enrichment analysis results of 82 candidate genes. **(A)** Venn diagram showing intersection of genes. **(B)** GO-BP enrichment network for candidate genes. **(C)** GO-MF enrichment network for candidate genes. **(D)** GO-CC enrichment network for candidate genes. **(E)** KEGG pathway enrichment diagram for candidate genes. **(F)** PPI network of candidate genes.

Next 82 candidate genes were obtained by intersecting DEGs1, DEGs2 and the key module genes (Figure 4A). GO analysis revealed that these candidate genes were significantly enriched in functions related to platelet-derived growth factor binding, ECM organization and basement membrane assembly, among others (Figures 4B–D). Furthermore, 22 KEGG pathways were found to be enriched, with the candidate genes predominantly involved in the ECM-receptor interaction, PI3K-Akt signaling, and focal adhesion pathways (Figure 4E). Within the PPI network, MMP14 was observed to interact with KPNA2, MAP1B, COL4A2, and other proteins (Figure 4F).

### 3.4 Robust predictive ability of the risk model

**FIGURE 5:**
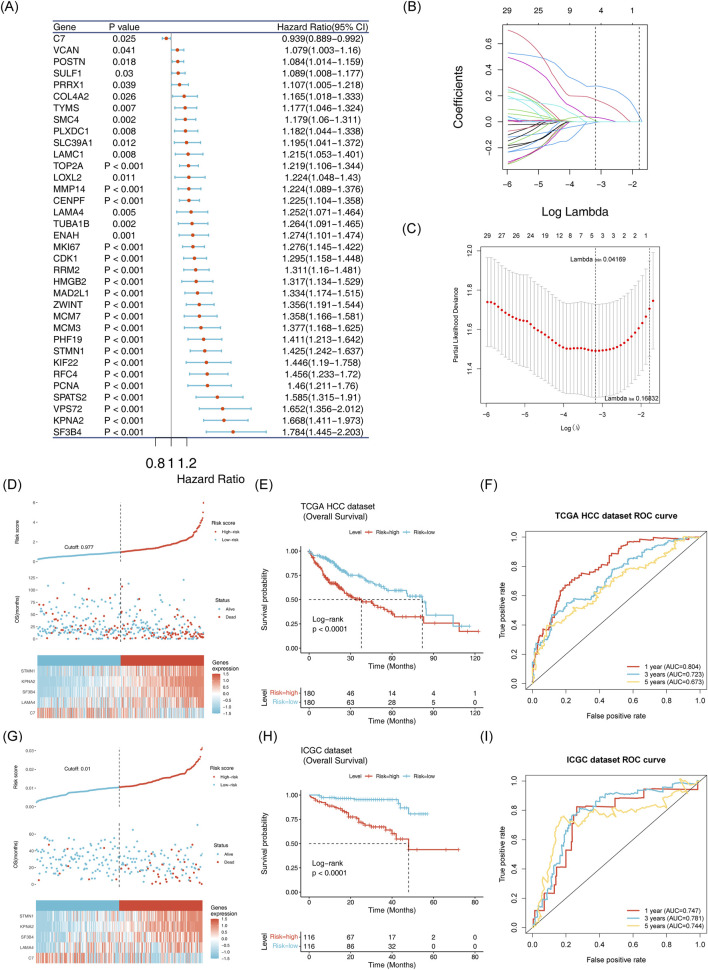
Establishment and validation of the risk model **(A)** Identification of 35 survival-related genes via univariate Cox regression analysis. **(B,C)**: Selection of five model genes using LASSO regression analysis. **(D)** Risk curve, scatter plot, and heatmap of model gene expression for high risk and low risk groups in the training set. **(E)** Survival curve for high-risk and low-risk groups in the training set. **(F)** ROC curve for the training set at 1, 3, and 5 years. **(G)** Risk curve, scatter plot and model gene expression heatmap for high risk and low risk groups in the ICGC-LIPI-JP dataset. **(H)** Survival curve for high risk and low risk groups oin the ICGC-LIPI-JP dataset.**(I)**: ROC curve for 1, 3, and 5 years survival in the ICGC-LIPI-JP dataset.

Based on the candidate genes, five biomarkers (LAMA4, C7, KPNA2, STMN1 and SF3B4) were identified through univariate Cox regression and LASSO analyses to construct a risk model (Figures 5A–C; Supplementary Table S2). Patients in the training set were stratified into high-risk and low-risk groups according to this model (Figure 5D). Survival rates were significantly lower in the high-risk group compared to the low-risk group (Figure 5E). The model demonstrated robust prognostic performance, with the area under the curve (AUC) values for 1, 3, and 5 years being 0.804, 0.723 and 0.673, respectively (Figure 5F). These results were validated using the independent validation set (Figures 5G–I).

### 3.5 The nomogram model had the capability for accurate prediction

**FIGURE 6:**
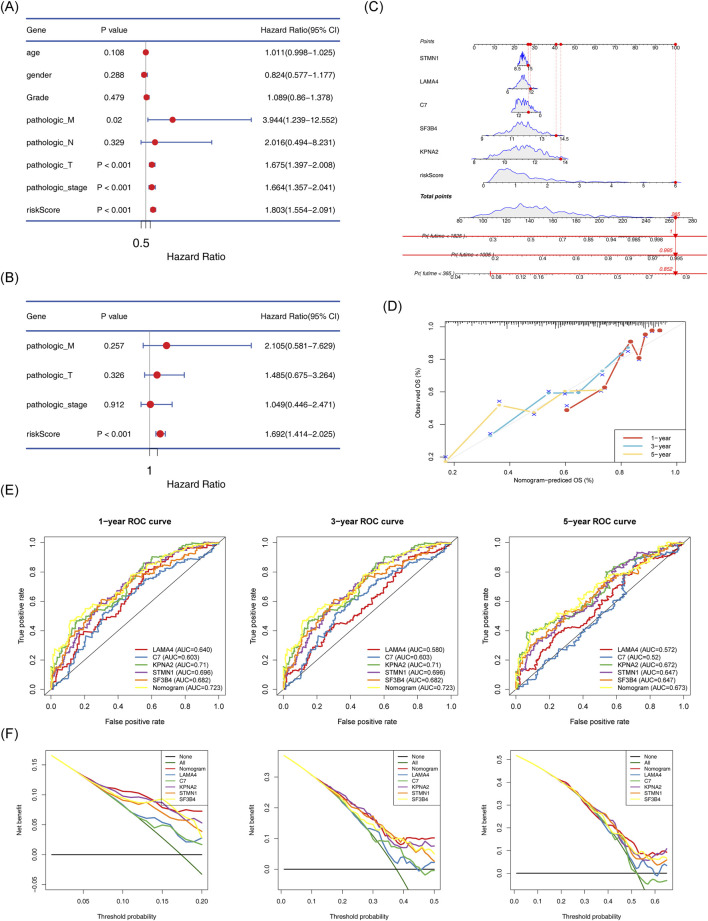
Establishment of the nomogram model and its evaluation. **(A)** Cox-independent prognostic analysis forest plot for TCGA-HCC. **(B)** Multifactor Cox-independent prognostic analysis forest plot for TCGA-HCC. **(C)** Nomogram predicting 1-, 3-, and 5-year survival. **(D)** Column chart and calibration curve predicting 1-, 3-, and 5-year survival. **(E)** ROC curve for the nomogram. **(F)** Decision curve analysis (DCA) for TCGA-HCC.

Risk scores, identified as independent prognostic factors, were calculated (Figures 6A,B; Supplementary Table S3). A nomogram integrating the five biomarkers was constructed (Figure 6C) The calibration curve indicated a close alignment with a slope of 1, suggesting good predictive accuracy (Figure 6D). The model exhibited AUC values greater than 0.6 for 1, 3, and 5-year survival predictions. Moreover, the model demonstrated superior efficiency compared to any single gene (Figures 6E, F).

### 3.6 Risk scores and survival differences among different clinical subgroups

**FIGURE 7:**
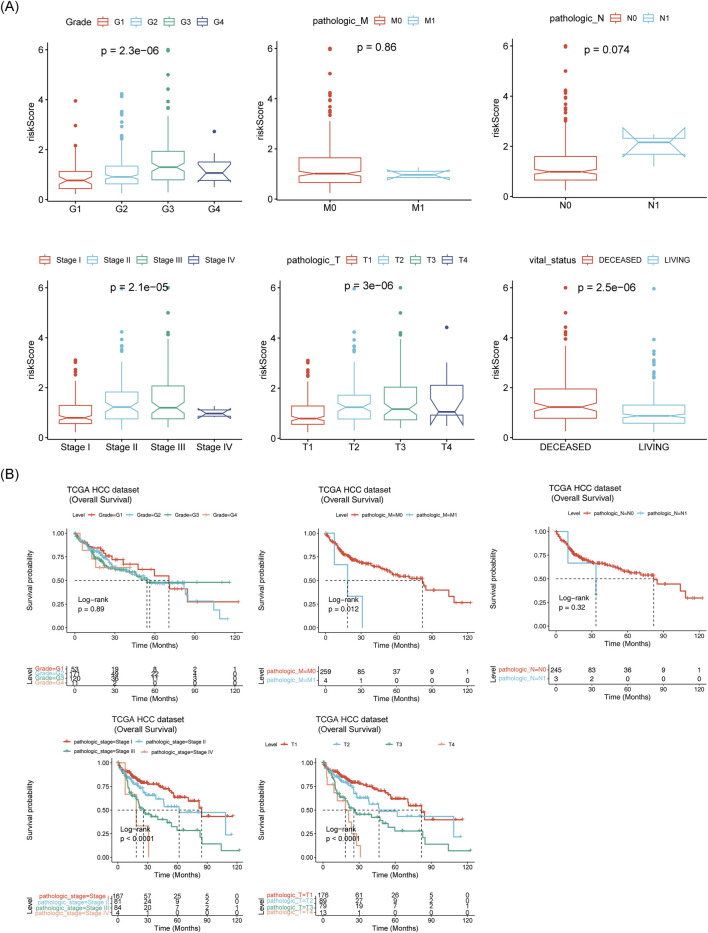
Correlation between prognostic model and clinical factors. **(A)** Box plots of risk scores across different clinical subtypes. **(B)** Survival analysis of different clinical subtypes.

Further analysis revealed significant differences between the two risk groups in terms of vital status, OS time, grade, pathologic T stage, and pathologic stage (Supplementary Table S4). The risk scores differed substantially across subgroups based on grade, pathologic stage, pathologic T stage, and vital status (Figure 7A). Survival differences were also observed within subgroups defined by pathologic-stage, pathologic T stage, and pathologic M stage (Figure 7B).

### 3.7 Exploration of the potential mechanisms of the 5 biomarkers

**FIGURE 8:**
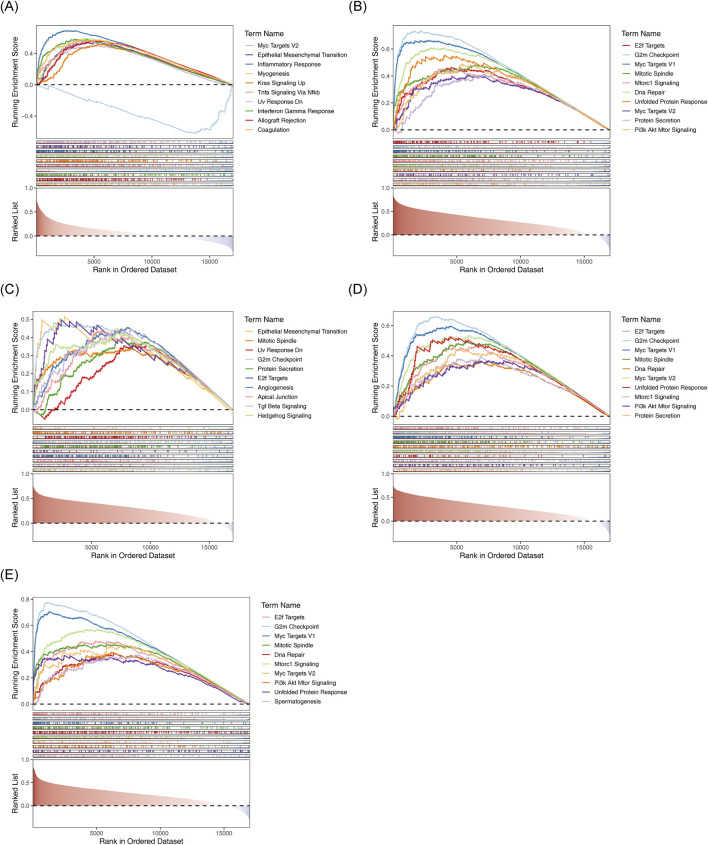
GSEA results for the five biomarkers. **(A)** C7-HALLMARK enrichment ridge plot. **(B)** KPNA2-HALLMARK enrichment ridge plot. **(C)** LAMA4-HALLMARK enrichment ridge plot. **(D)** SF3B4-HALLMARK enrichment ridge plot. **(E)** STMN1-HALLMARK enrichment ridge plot.

C7 was significantly enriched in pathways related to EMT, inflammatory response, and upregulation of KRAS signaling (Figure 8A). KPNA2, LAMA4, SF3B4, and STMN1 were enriched in processes such as the G2M checkpoint and mitotic spindle formation (Figures 8B–E).

### 3.8 Dissection of the immunological landscapes across risk groups

**FIGURE 9:**
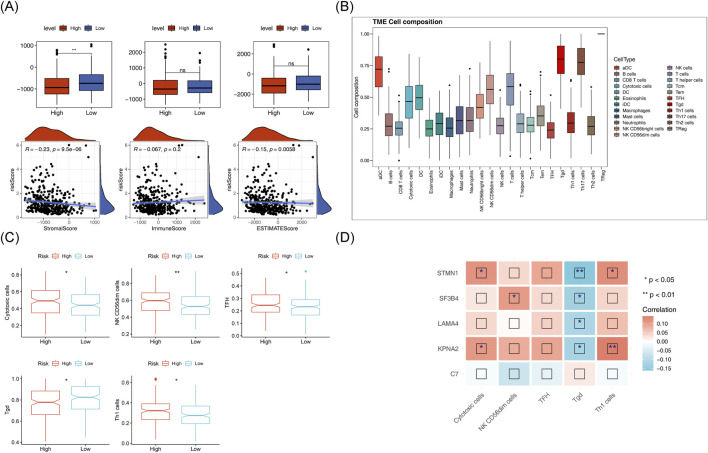
Immunoanalysis of high and low risk groups. **(A)** ESTIMATE differences between high and low risk groups and their correlation with risk scores. **(B)** Histogram of immune cell proportion (score). **(C)** Box plot of differential immune cell proportions (scores) in high and low-risk groups **(D)** Heat map of correlation between model genes and differential immune cells.

**FIGURE 10:**
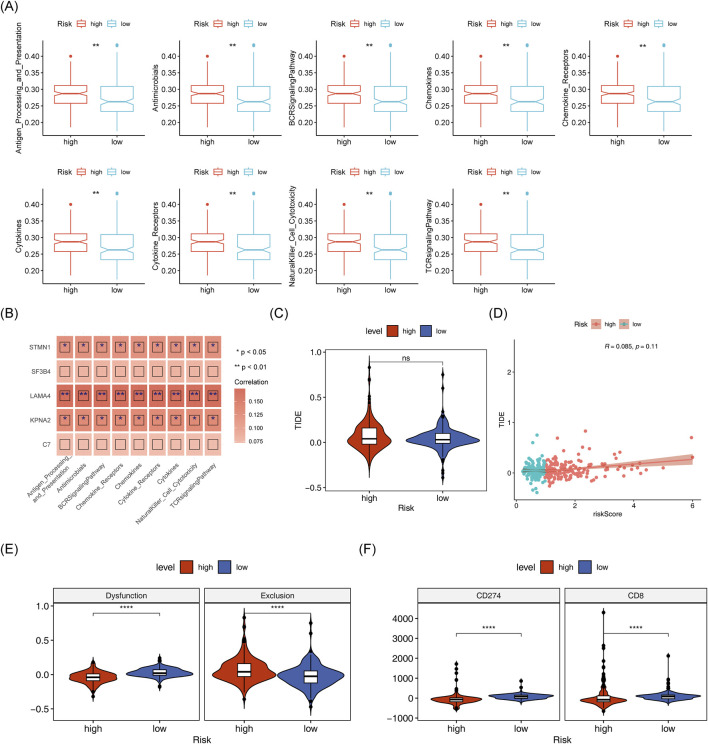
Analysis of immune-related pathways in high and low-risk groups. **(A)** Box plot of differential immune pathway scores in high and low-risk groups. **(B)** Heatmap of correlation between model genes and immune pathway scores. **(C,D)**: TIDEscores and their correlation with risk scores in high and low-risk group. **(E,F)**: Differences in immune dysfunction, immune rejection scores, CD274, and CD8 expression between high-risk groups.

Matrix scores significantly differentiated between the two risk groups andwere negatively correlated with the risk score (Figure 9A). Immune cell infiltration was evident across all HCC samples (Figure 9B). Cytotoxic cells, TFH, NK CD56dim cells, Tgd and Th1 cells showed clear differences between the two risk groups (Figure 9C). A negative correlation was found between Tgd cell abundance and STMN1expression, while a significant positive correlation was observed between Th1 cell abundance and KPNA2 expression (Figure 9D). Immune-related pathway scores for all 9 immune pathways showed significant differences between the two risk groups, including pathways like antimicrobials, BCR signaling, and chemokines (Figure 10A). Additionally, KPNA2, LAMA4, and STMN1 were positively correlated with these immune-related pathways (Figure 10B). However, the TIDE score did not show significant differences between the two groups nor any correlation with the risk score (Figures 10C, D). Notable differences were also observed in immune dysfunction, immune rejection scores, CD274, and CD8 expression between the two risk groups (Figures 10E,F).

### 3.9 Unraveling the mutational patterns in two risk groups

**FIGURE 11:**
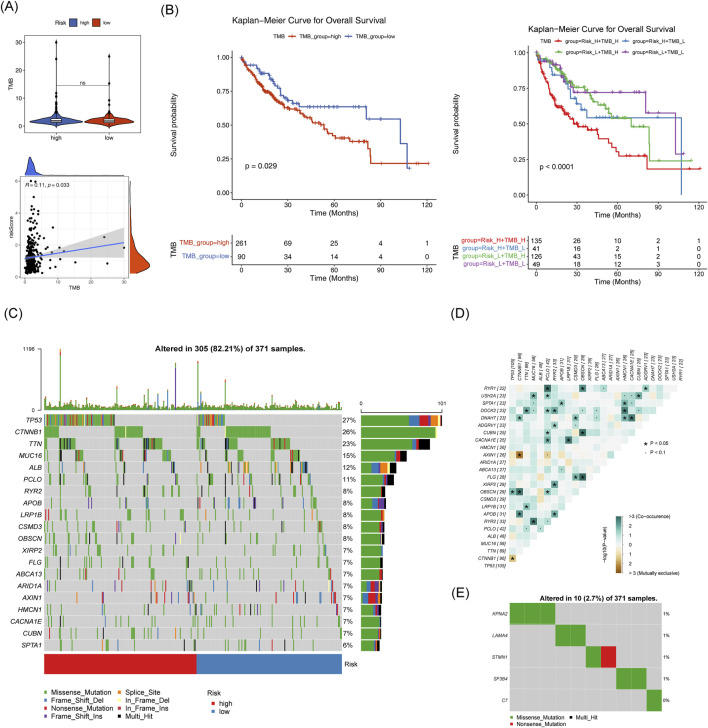
Mutation analysis in high and low-risk groups. **(A)** TMB box plot and scatter plot showing correlation with risk scores for high and low-risk groups. **(B)** TMB box plot and scatter plot showing correlation with risk scores for high and low-risk groups. **(C)** Top 20 mutated genes in high and low-risk groups. **(D,E)**: Correlation of mutated genes.

TMB did not show a significant difference between the two risk groups, however, it exhibited a strong correlation with the risk score (*p* = 0.033) (Figure 11A). TCGA-HCC individuals were divided into H-TMB and L-TMB groups based on the optimal threshold. These patients were further subdivided into four subgroups: H-TMB with High Risk, H-TMB with Low Risk, L-TMB with High Risk, and L-TMB with Low Risk. Significant survival differences were observed among these four groups (*p* < 0.001) (Figure 11B). Additionally, Figure 11C, D shows the top 20 mutated genes in the two risk groups, and the interrelationships among them. Notably, the mutation types of the five biomarkers predominantly consisted of missense mutations (Figure 11E).

### 3.10 The IC50 values of 23 drugs differed significantly between the two groups

**FIGURE 12:**
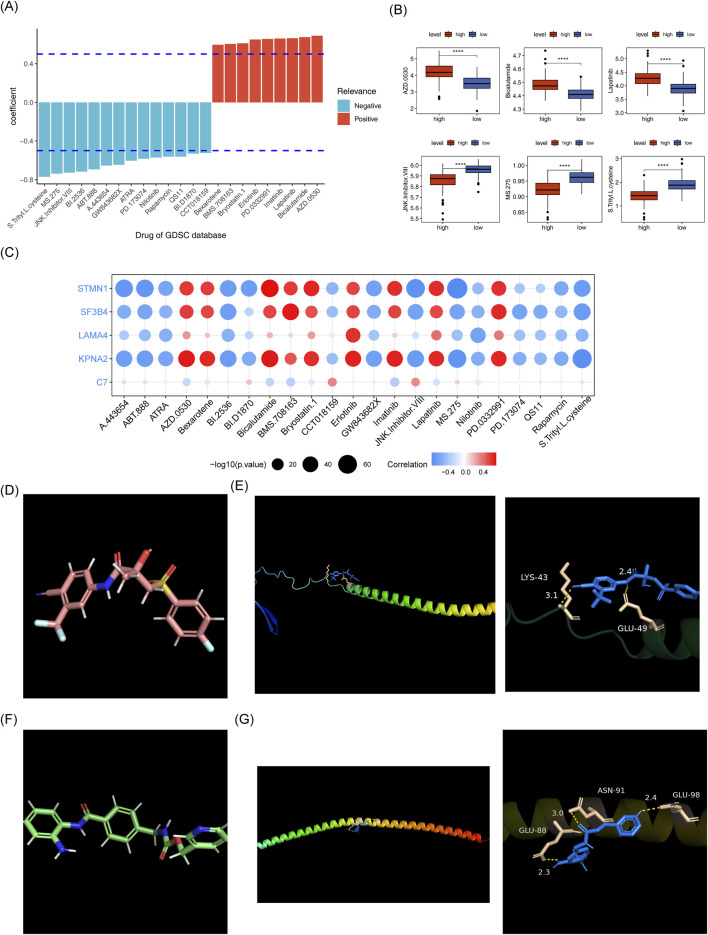
Risk score and drug sensitivity analysis. **(A)** Histogram of drug sensitivity related to risk scores. **(B)** Box plot of IC50 values for drugs in the high and low-risk groups. **(C)** Heatmap of correlation between model genes and differential drugs. **(D)** Schematic diagram of the 3D conformerr structure of Bicalutamide. **(E)** PDB ID AF_AFP16949F1 figure showing docking results with Bicalutamide. **(F)** Schematic diagram of the 3D conformerr structure of MS.275. **(G)** PDB ID AF_AFP16949F1 connection results with MS.275.

A total of 14 drugs exhibited a significant negative correlation with the risk score (cor < −0.5, *p* < 0.05), while 9 drugs showed a strong positive correlation with the risk score (cor >0.5, *p* < 0.05) (Figure 12A). The IC50 of 23 drugs varied significantly between the two risk groups, as shown in Figure 12B. Among these, the IC50 values of the top 3 drugs with the strongest positive and negative correlations (AZD.0530, Bicalutamide, Lapatinib, JNk.intervener.VIII, MS.275 and S. Treyl.l.costeine) were analyzed. KPNA2, STMN1, and Bicalutamide exhibited a notably strong positive correlation, while MS-275 showed a significant negative correlation (Figure 12C). The drug-protein pairs with the strongest positive (STMN1 and Bicalutamide) and negative (STMN1 and MS.275) correlations were selected for molecular docking. The 3D conformer structure of Bicalutamide and the crystal structure of PDB ID AF_AFP16949F1 corresponding to STMN1 were downloaded, and molecular docking was performed (binding energy = −5.0 kcal/mol). Hydrogen bonding interactions were observed between Bicalutamide molecules and LYS-43, GLU-49 of STMN1 (Figures 12D, E). For the negative correlation pair, the 3D conformer structure of MS.275 and the crystal structure of STMN1 (PDB ID AF_AFP16949F1) were used, with a binding energy of −5.8 kcal/mol, Hydrogen bonding was detected between MS.275 molecules and ASN-91, GLU-98, and GLU-88 of STMN1 (Figures 12F, G).

### 3.11 Specific expression patterns of biomarkers

**FIGURE 13:**
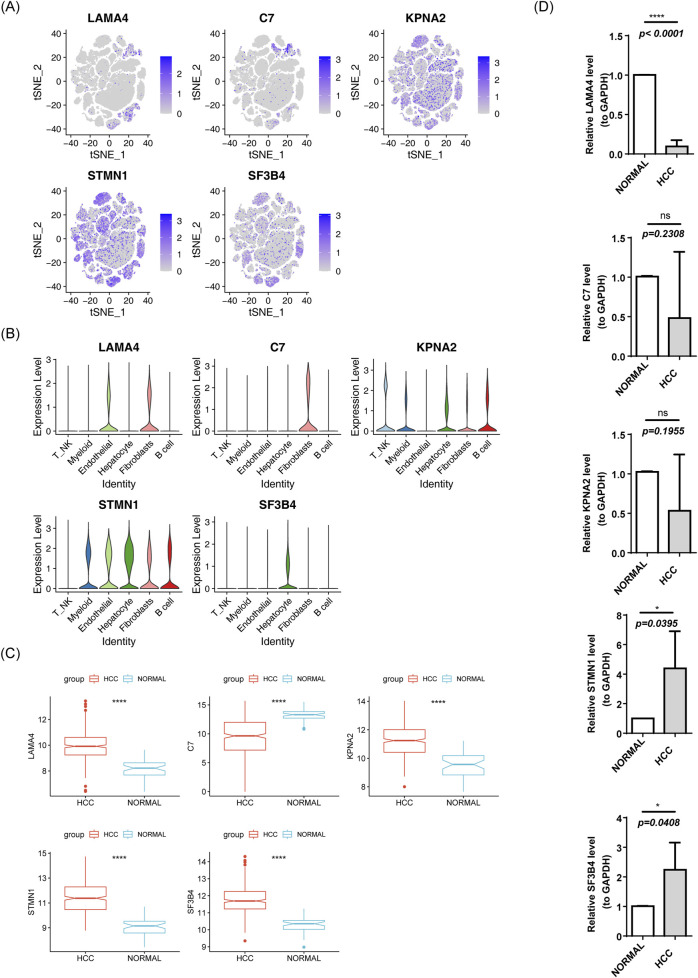
Expression of risk model genes in each cell subpopulation. **(A)** t-SNE clustering of prognostic model genes. **(B)** Violin plot of prognostic model gene expression. **(C)** Box plot of gene expression in the TCGA-HCC prognostic model. **(D)** RT-qPCR results.

The expression levels of biomarkers in each cell subpopulation were visualized (Figure 13A). LAMA4 showed higher expression in Endothelial cells and Fibroblasts, C7 was higher in Fibroblasts, KPNA2 exhibited higher expression in T_NK and B cells, and STMN1 and SF3B4 were more highly expressed in Hepatocytes (Figure 13B). In the TCGA-HCC and GSE76427 datasets, except for C7, the other biomarkers were upregulated in HCC samples (Figure 13C; Supplementary Figure S2). To validate these findings, RT-qPCR was performed. SF3B4 and STMN1 were significantly upregulated in the HCC group (*p* = 0.0408, *p* = 0.0395, respectively), which was consistent with the TCGA-HCC dataset results. However, no significant differences were found in the expression of KPNA2 and C7 between the two groups (all *p > 0.05*), and LAMA4 expression was higher in the control group (*p* < 0.001), possibly due to sample heterogeneity (Figure 13D).

## 4 Discussion

HCC ranks among the top five causes of cancer-related mortality, with steadily rising incidence (Hartke et al., 2017). The prognosis for HCC is poor, and the recurrence rate remains high. Even after hepatectomy, patients are highly susceptible to relapse within 6 months (Yasuda et al., 2024). Therefore, early diagnosis and intervention are critical. Growing evidence emphasizes the role of EMT as a key driver of cancer metastasis (Wang et al., 2022). Additionally, anoikis, a specific form of programmed cell death, has been identified as a key factor in cancer progression (Lu et al., 2015). The interplay between EMT and anoikis has emerged as a focal point of research in recent years.

This study involved a comprehensive screening of genes associated with both EMT and anoikis, leading to the identification of five biomarkers, namely, LAMA4, C7, KPNA2, STMN1 and SF3B4. LAMA4 induces CD8^+^ T cell senescence in HCC via the ITGA6 receptor-mediated DNA damage signaling pathway. Targeting LAMA4 suppresses malignant progression and enhances the efficacy of anti-PD-1 therapy (Zhang et al., 2025). LAMA4 interacts with integrin receptors, activating focal adhesion kinase (FAK) and Src family kinases (SFKs), which regulat cellular adhesion and migration, thereby contributing to the EMT process (Speisky et al., 2012). LAMA4, through its binding to integrins, phosphorylates FAK and activates AKT, inhibiting pro-apoptotic proteins, This mechanism enables tumor cells to resist anoikis and metastasize. Targeting. LAMA4 may enhance anoikis sensitivity and inhibit metastasis (Liu G. et al., 2024). C7 induces cell membrane perforation and apoptosis by forming the membrane attack complex (MAC) in collaboration with other complement components. It targets tumor cells with low expression of surface complement regulatory proteins. Reduced C7 mRNA levels are associated with advanced cancer stages and higher tumor grades in patients with HCC (Qian et al., 2022). C7-mediated MAC formation directly induces tumor cell lysis and suppresses the EMT process (Xu et al., 2022). Additionally, C7-mediated MAC formation enhances membrane damage in cells detached from the ECM, synergizing with anoikis to eliminate tumor cells (Wong et al., 2021). KPNA2 is overexpressed in various cancers and correlates with poor prognosis, particularly in HCC where it is linked to immunological infiltration and may play a role in cell cycle regulation (Pan et al., 2023). Xia et al. demonstrated that KPNA2 has oncogenic functions, and may modulate tumor progression through EMT. Furthermore, riboflavin α2 may influence the malignant phenotype of cells by binding to the KPNA2 promoter, thereby regulating EMT (Xia and Ma, 2022). STMN1, a microtubule-associated phosphoprotein, plays a pivotal role in HCC progression by modulating DNA methylation, regulating m6A RNA modification, and influencing disease-related immune responses, STMN1 serves as a key biomarker for HCC diagnosis and prognosis (Zhang E. D. et al., 2022). Liu et al. demonstrated that miR-221 promotes TGFβ1-induced EMT in human bladder cancer cells by targeting STMN1, positioning it as a promising therapeutic target for metastasis (Liu et al., 2015). Additionally, Huang et al. found that SF3B4 regulates HCC proliferation and apoptosis through alternative splicing and interactions with TRIM28 and SETD5, underscoring its potential as a therapeutic target for HCC (Huang et al., 2025). Furthermore, SF3B4 promotes cell migration and invasion through Twist1-mediated mechanisms. As Twist1 is a key transcription factor driving EMT, SF3B4 is believed to be closely associated with EMT regulation (Yang et al., 2023). All five biomarkers identified in our study have been implicated in HCC, EMT, and anoikis resistance, with their roles in regulating HCC progression aligning with previous findings.

Compared to existing prognostic tools for HCC, such as the TNM staging system, Child-Pugh classification, and the China Liver Cancer (CNLC) staging system, the proposed prognostic model and nomogram offer superior predictive potential. The model allows for identification of high-risk patients, even those in early stages according to the TNM staging, facilitating proactive surveillance and intervention before disease progression. Furthermore, it provides more personalized insights for clinical decision-making, extending beyond tumor anatomical characteristics. The model’s clinical applicability is also significant. It can be implemented in clinical practice to stratify patients with HCC into risk categories, helping clinicians identify high-risk individuals. This supports the adoption of more aggressive treatment strategies, such asenhanced follow-up and earlier adjuvant therapies. For high-risk patients, surgical resection combined with postoperative adjuvant therapy may be more appropriate, while low-risk patients may benefit from conservative approaches like local ablation. Adjusting follow-up frequency and diagnostic protocols based on the predicted risk levels would optimize healthcare resource allocation. However, the model has several limitations, The sample size may be limited and the samples may primarily originate from a single institution or specific regions, which could affect the model’s generalizability.

Gene expressionanalysis across different cell types indicated that LAMA4 and STMN1 were highly expressed in endothelial cells and fibroblasts, while C7 was predominantly expressed in fibroblasts. Endothelial cells are primarily involved in tumor angiogenesis, and fibroblasts play a pivotal role in promoting fibrosis or tumor growth, indicating that these three biomarkers significantly contribute to tumor progression. KPNA2 showed high expression in B cells and T cells, indicating its potential relevance to immune regulation. Moreover, SF3B4 was highly expressed in hepatocytes, implying a potential role in hepatocyte transformation (Xie and Wang, 2025).

Furthermore, GSEA enrichment analysis revealed that C7 was enriched in EMT, while the other four biomarkers were eassociated with the G2/M checkpoint and mitotic spindle pathways. Research indicates that the MAC formed by C7 can directly induce tumor cell lysis and inhibit EMT (Xu et al., 2022), thus reducing metastasis and progression of HCC, which supports our findings. DNA damage can trigger G2/M arrest while inhibiting integrin-FAK signaling, thereby reducing cellular adhesion capacity and increasing sensitivity to anoikis (Vaz et al., 2021). Dysregulation of core regulatory proteins at the G2/M checkpoint can drive premature mitosis without completing DNA repair, often leading to genomic instability and promoting HCC progression. Notably, Coptisine-mediated downregulation of E2F7 disrupts G2/M-phase protein functions, providing a potential therapeutic strategy for HCC (Wang et al., 2024). EMT has been shown to enhance tumor mitosis within mechanical microenvironments by facilitating spatial accommodation for mitotic spindle formation, thereby promoting cancer cell proliferation (Hosseini et al., 2020). Zhang et al. constructed a risk assessment model based on mitotic spindle assembly-related genes, which not only accurately predicts HCC prognosis but also identifies novel therapeutic targets for treatment (Zhang et al., 2024). Moreover, Spearman correlation analysis of immune pathway scores and prognostic model genes revealed significant positive correlations between KPNA2, LAMA4, and STMN1 with nine immune-related pathways. These findings suggest that these genes may influence HCC development through these pathway.

Numerous studies highlight the significant role of immune cells in influencing the progression of HCC (Hao et al., 2021). In the present study, immunoanalyses of the high-risk and low-risk groups revealed notable differences in immune cell populations and immune-related pathways. NK cells, essential components of the innate immune system’s cytotoxic lymphocytes, play a critical role in eliminating viral infections and cancer cells. Dysfunction in NK cells is strongly associated with HCC progression (Sajid et al., 2022). Zhang et al. demonstrated through genetic analysis of patients with HCC, that those with poor survival outcomes had a higher number of NK and Th1 cells (Chang et al., 2023), which aligns with our results showing increased Th1 cell presence in the high-risk group. Chemokines and their receptors also significantly contribute to HCC progression, and remain a focal point of research. High expression of chemokine has been linked to both HCC and colorectal liver metastasis (Rubie et al., 2006), and our findings of elevated chemokine expression in the high-risk group support this notion. Additionally, while TIDE scores showed no significant differences between the two risk groups, there were notable discrepancies in immune dysfunction and immune exclusion scores. This suggests that the high-risk group exhibits a greater proportion of immune cells with compromised functionality, reducing their ability to effectively target and eliminate cancer cells even when present within the tumor. Furthermore, it indicates that a larger proportion of immune cells in the high-risk group are excluded from the tumor microenvironment, preventing them from infiltrating the tumor core and launching an immune attack. These findings can offer insight into patient responses to immunotherapy, which is consistent with findings reported by Wang et al. (Zhang E. D. et al., 2022). Xie et al. also utilized relevant indicators to identify lncRNA features associated with m6A modification and ferroptosis to predict the immune efficacy in HCC (Xie et al., 2022).

Drug susceptibility analysis highlighted a significant inverse correlation between the IC50 values of 14 drugs and the risk score, while a strong positive relationship was found between the IC50 values of nine drugs and the risk score. The most significantly correlated drugs included AZD.0530, bicalutamide, Lapatinib, JNk.intervener.VIII, MS.275, and S. Treyl.L.osteine with the first three showing a positive correlation and the latter three a negative correlation. Bicalutamide is used to treat prostate cancer (Jia and Spratt, 2022), and Lapatinib is widely employed in the treatment of breast cancer (Bilancia et al., 2007). Chidamide, a structural analog of MS-275, may inhibit HCC cell growth by upregulating p21, inducing cell cycle arrest (Wang et al., 2012). Our findings suggest that drugs negatively correlated with the risk score may be more effective in treating patients in the high-risk group, providing valuable insights for developing personalized therapeutic strategies. Furthermore, a binding energy of −5.8 kcal/mol between the 3D conformer structure of MS.275 and the crystal structure of STMN1 (PDB ID AF_AFP16949F1) indicates a stronger affinity, suggesting that MS-275 may be a promising therapeutic agent.

PCR analysis revealed inconsistencies in the expression levels of the five biomarkers, with only SF3B4 and STMN1 showing positive results. This variation is likely due to sample heterogeneity. Differences in sample sources and clinical characteristics, such as age, gender, and tumor stage, can contribute to discrepancies in gene expression. Additionally, the expression of biomarkersmay vary across different patients with HCC at different stages. Discrepancies in RNA extraction efficiency, reverse transcription, and amplification conditions may also contribute to these inconsistencies.

In recent years, research on biomarker-based models for HCC has surged. Notably, Mu et al. developed a non-invasive nomogram based on interleukin-41 (IL-41) to predict poor prognosis in HCC, achieving promising results in forecasting recurrence and mortality (Mu et al., 2025). Saeed et al. identified RACGAP1 and MKI67 as potential prognostic biomarkers for HBV/HCV-associated HCC mediated by lactylation, thus pioneering new avenues for immune-targeted therapies (Saeed et al., 2025). Among the many multi-omics-based prognostic models, Bai et al. established a biological model closely associated with tumor prognosis in HCC through pan-cancer multi-omics analysis of monocyte-macrophage differentiation (MMD) (Bai et al., 2025). Additionally, research focusing on therapeutic interventions targeting EMT and anoikis has been expanding. For example, emodin reverses sorafenib resistance in HCC by suppressing EMT through inhibition of the Akt signaling pathway (Wang and Zhang, 2025). Mesoporous cerium oxide nanozymes also effectively inhibit HCC metastasis by inducing anoikis resistance (Wang et al., 2025). Furthermore, in the adjuvant treatment of HCC, monotherapy with immune checkpoint inhibitors (ICIs) demonstrates the best safety profile, outperforming dual ICIs and combinations of targeted therapy and immunotherapy, providing valuable insights for future therapeutic strategies (Wu et al., 2025). These innovations in model and treatment strategies for HCC are driven by extensive research, Iand pave the way for more refined and comprehensive diagnostic and therapeutic approaches.

However, this study also has certain limitations. Firstly, the sample size for the bioinformatic analysis was limited, which may affect the generalizability of the results. Additionally, the specific mechanisms of action of the biomarkers in HCC have not been experimentally validated. Therefore, we plan to integrate more public databases and clinical samples in the future to strengthen the statistical power and validate the reliability of our findings. Concurrently, we will conduct *in vitro* (e.g., knockdown/overexpression in cell lines) and *in vivo* (e.g., mouse models) experiments, utilizing techniques such as immunohistochemistry (IHC) and Western blotting (WB), to investigate the specific functions and regulatory pathways of the biomarkers in HCC.

## 5 Conclusion

This study integrated the screening of EMTRGs and ARGs, identified identify five biomarkers and developed a risk model. The model was designed to explore the potential prognostic implications of these biomarkers in HCC and investigate their associations with immune resistance and relevant pathways. The findings open new avenues for understanding the molecular mechanisms and prognostic predictions of HCC. However, a deeper understanding of the functional mechanisms and clinical relevance of these genes is still required and warrants further research. Future studies will aim to elucidate their roles with greater precision.

## Data Availability

The datasets [TCGA-HCC, ICGC-LIPI-JP and GSE149614] used in this study are available in the following databases: [TCGA database, ICGC database, and GEO database] [http://cancergenome.nih.gov; https://dcc.icgc.org/releases/current/Projects/LIRI-JP; https://www.ncbi.nlm.nih.gov/geo/].
