# Supplementary figures and images for: Identification of prognostic biomarkers related to epithelial-mesenchymal transition and anoikis in hepatocellular carcinoma using transcriptomics and single-cell sequencing
Source: Front Cell Dev Biol. 2025 Jun 19;13:1600546. doi: 10.3389/fcell.2025.1600546 (PMC12223776; doi:10.3389/fcell.2025.1600546)

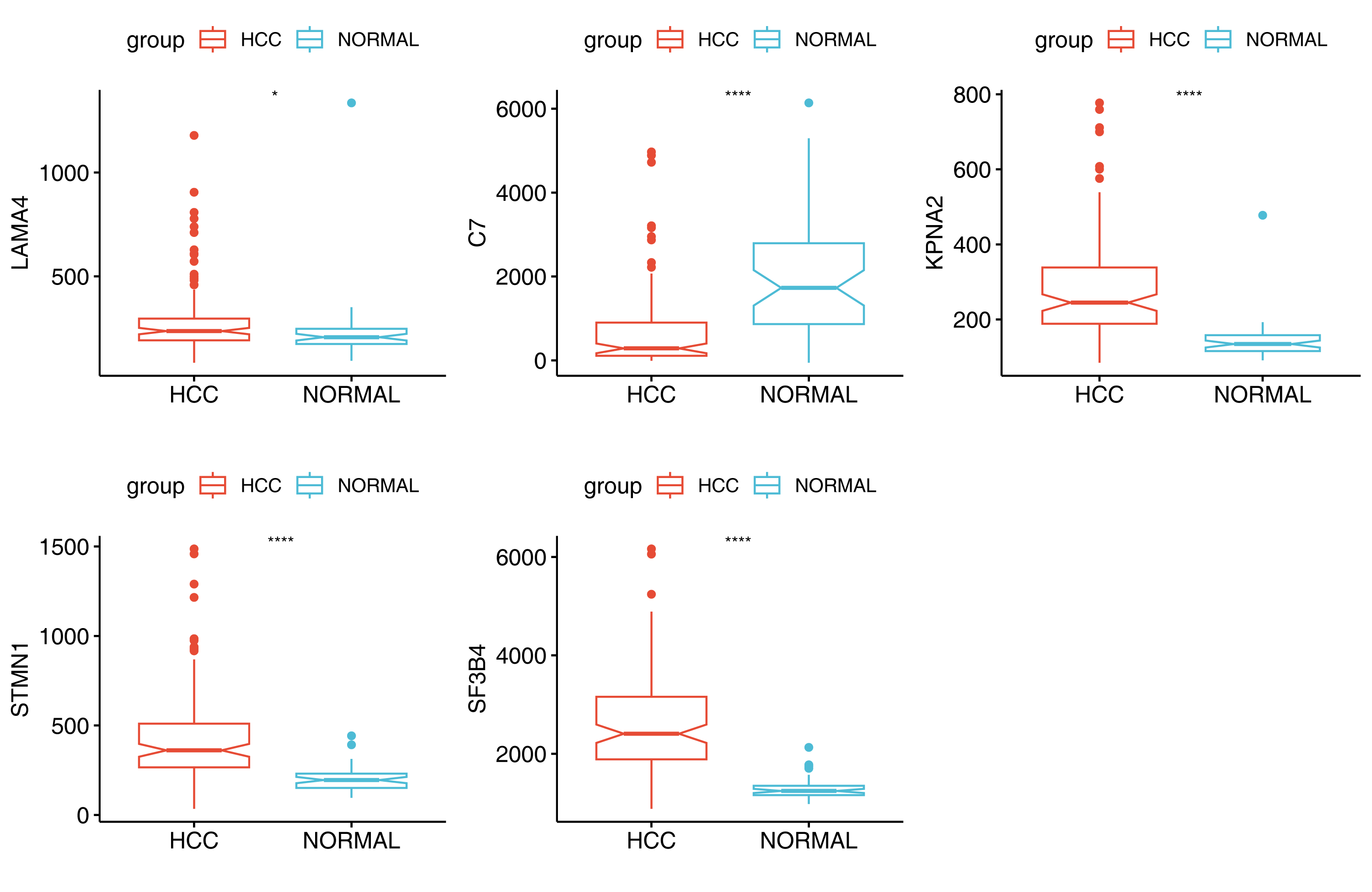

Supplement: Supplementary file 3 [file Image3.TIF]

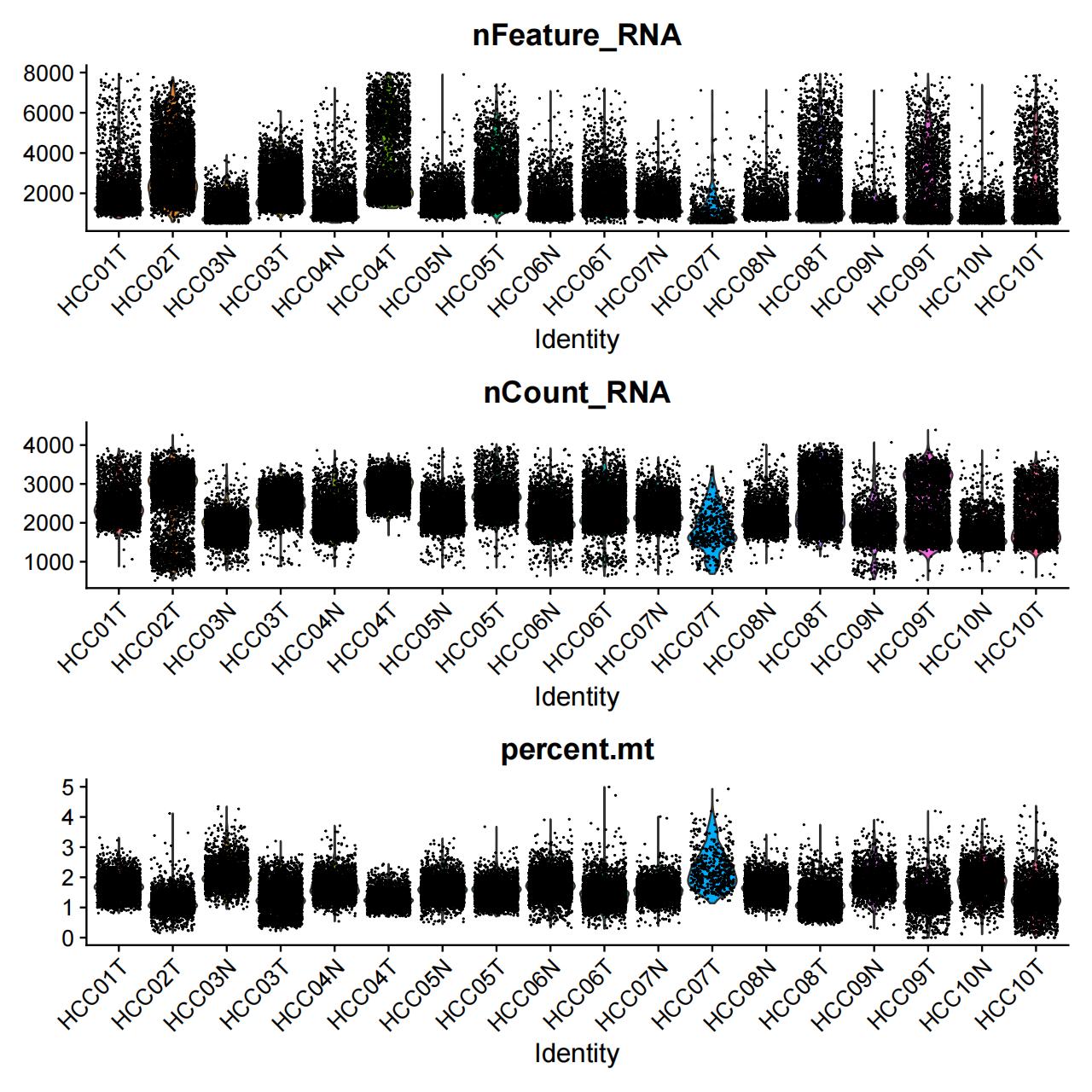

Supplement: Supplementary file 4 [file Image2.JPEG]

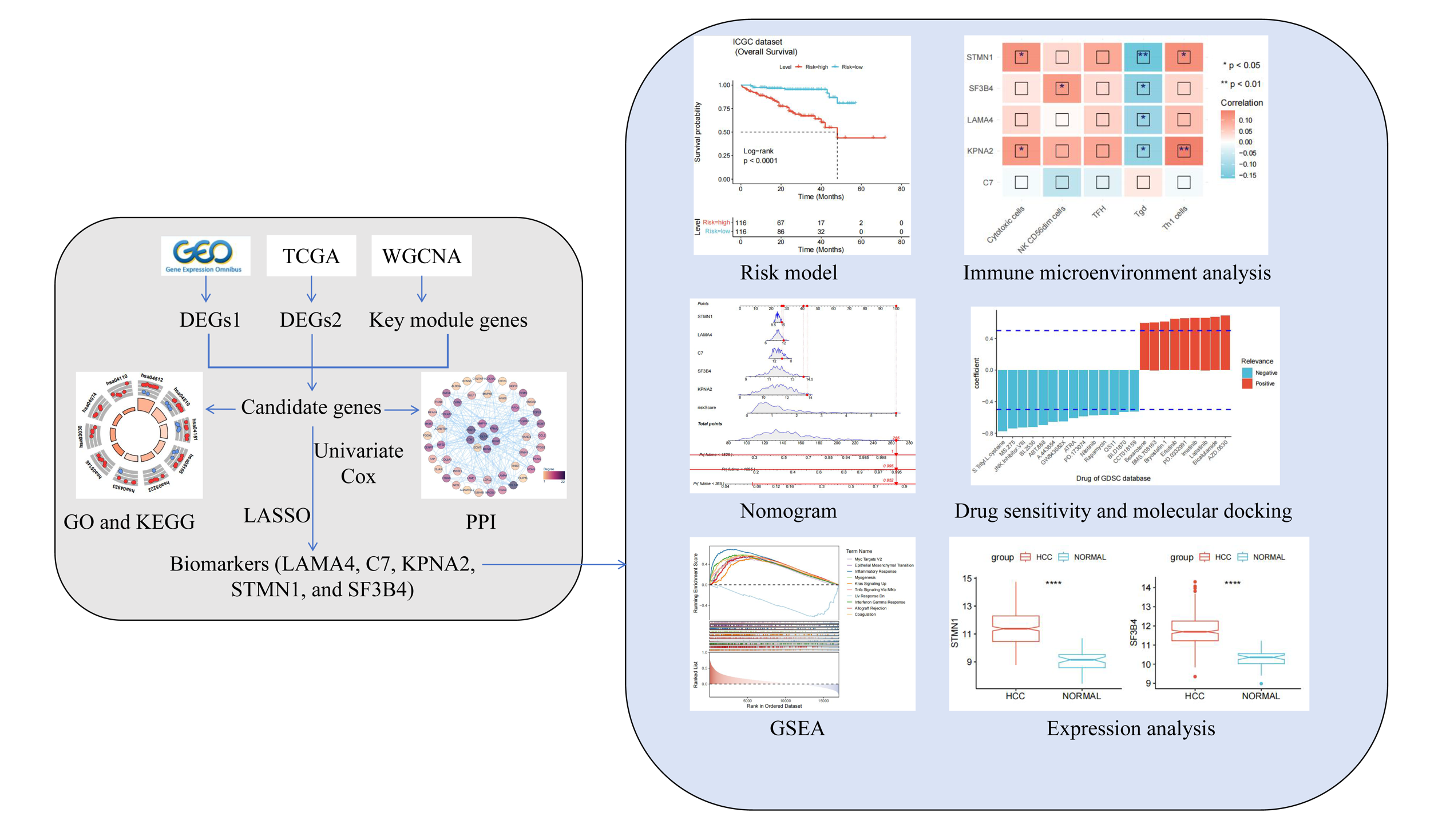

Supplement: Supplementary file 5 [file Image1.TIF]
